# Immunity and Nutrition: The Right Balance in Inflammatory Bowel Disease

**DOI:** 10.3390/cells11030455

**Published:** 2022-01-28

**Authors:** Bartolo Tamburini, Marco Pio La Manna, Lidia La Barbera, Leila Mohammadnezhad, Giusto Davide Badami, Mojtaba Shekarkar Azgomi, Francesco Dieli, Nadia Caccamo

**Affiliations:** 1 Department of Biomedicine, Neurosciences and Advanced Diagnostic (Bi.N.D.), University of Palermo, 90127 Palermo, Italy; bartolo.tamburini@unipa.it (B.T.); leila.mohammadnezhad@unipa.it (L.M.); giustodavide.badami@unipa.it (G.D.B.); mojtaba.shekarkarazgomi@unipa.it (M.S.A.); francesco.dieli@unipa.it (F.D.); nadia.caccamo@unipa.it (N.C.); 2 Central Laboratory of Advanced Diagnosis and Biomedical Research (CLADIBIOR), University of Palermo, via del Vespro 129, 90127 Palermo, Italy; 3 Rheumatology Section, Department of Health Promotion, Mother and Child Care, Internal Medicine and Medical Specialties, University of Palermo, Piazza delle Cliniche, 2, 90110 Palermo, Italy; lidia.labarbera@unipa.it

**Keywords:** immune bowel disease, cytokines, dysbiosis, polyunsaturated fatty acids

## Abstract

Inflammatory bowel disease (IBD) is an increasingly urgent medical problem that strongly impairs quality of life for patients. A global rise in incidence has been observed over the last few decades, with the highest incidence rates recorded in North America and Europe. Still, an increased incidence has been reported in the last ten years in newly industrialized countries in Asia, including China and India, both with more than one billion inhabitants. These data underline that IBD is an urgent global health problem. In addition, it is estimated that between 20% and 30% of IBD patients will develop colorectal cancer (CRC) within their lifetime and CRC mortality is approximately 50% amongst IBD patients. Although the exact etiology of IBD is still being defined, it is thought to be due to a complex interaction between many factors, including defects in the innate and adaptive immune system; microbial dysbiosis, i.e., abnormal levels of, or abnormal response to, the gastrointestinal microbiome; a genetic predisposition; and several environmental factors. At present, however, it is not fully understood which of these factors are the initiators of inflammation and which are compounders. The purpose of this review is to analyze the complex balance that exists between these elements to maintain intestinal homeostasis and prevent IBD or limit adverse effects on people’s health.

## 1. Introduction

A global rise in incidence of IBD has been observed over the last decades, with the highest incidence rates recorded in high income countries. Still, an increased incidence has been reported in the last ten years in newly industrialized countries in Asia, including China and India, both with more than one billion inhabitants [1,2].

The gastrointestinal tract is the most sophisticated and complex immune organ of the body. The dynamic crosstalk between intestinal epithelial cells (IECs), the microbiome, and immune cells is crucial for the maintenance of intestinal homeostasis [3]. Its breakdown can lead to the onset of inflammatory bowel diseases (IBD), chronic relapsing diseases characterized by intestinal inflammation, and epithelial damage, whicharise in genetically susceptible people exposed to environmental risk factors [4,5]. There are two forms of IBD: Crohn’s disease (CD) and ulcerative colitis (UC) [6]. CD, characterized by transmural inflammation, affects the gastrointestinal tract, small intestine, and proximal colon, while UC, characterized by superficial inflammation in the mucosa and submucosa, is restricted to the rectum, colon, and cecum. Both disorders are associated with specific symptoms, such as diarrhea, abdominal cramps, weight loss, anemia, significant morbidity, and extraintestinal manifestations, including arthritis, dermatological symptoms, kidney stones, osteopenia or osteoporosis, vitamin deficiencies, and liver disease, such as primary sclerosing cholangitis [7].

The etiology of IBD is still being defined, but many different genetic, immune, microbial, and environmental factors contribute, which is nutrition. This latter factor probably exerts a more significant influence on IBD development: several studies have shown that a diet rich in proteins and unsaturated fats and low in fiber derived from fruits and vegetables can trigger a pro-inflammatory response in susceptible individuals [8]. In fact, at the genetic level, various mutations have been identified in more than 200 genes that encode or modulate protein expression on the regulation of the immune system in patients with IBD. This finding suggests that IBD patients can have a genetic background that affects the immune system, making them more likely to develop inflammatory diseases. For example, linkage studies showed the association with a locus on chromosome 16q12, known as nucleotide-binding oligomerization domain-containing protein 2/caspase activation recruitment domain (NOD2/CARD15). In particular, homozygosity at this locus involves an approximately 40-fold increased risk of CD, while heterozygosity is associated with a 2–4-fold risk. NOD2, predominantly expressed in macrophages, Paneth cells, dendritic cells, IECs, and T lymphocytes, recognizes the muramyl peptide in bacterial peptidoglycans of gram-positive and gram-negative. In addition, NOD2 activates the NF-κB pathway, regulating the secretion of proinflammatory and protective molecules involved in intestinal homeostasis. It also allows the recruitment of ATG16L to start the autophagic process. Specific mutations of NOD2 (Arg702Trp, Gly908Arg, 1007fs) cause a defective binding with the muramyl dipeptide with consequent alteration of NF-κB activation. This altered pathway increases the number of bacteria in the lumen, reduces elimination of pathogens, and inhibits the levels of antimicrobial peptides, such as defensins that allow the expression of NOD2 in Paneth cells. Thus, mutations in NOD2 and an ongoing inflammatory process can be decisive in causing the intestinal barrier breakdown and lead to a possible dysbiosis [8,9]. Other studies have shown a correlation between endoplasmic reticulum stress or the unfolded protein response (UPR) and IBD [10,11,12]. As a matter of fact, in subjects suffering from IBD, there is an increase in the stress of the endoplasmic reticulum in the intestinal epithelium, leading to an accumulation of unfolded or misfolded proteins in Paneth cells. [3,13,14,15,16]. These changes affect the immune response and can be decisive for triggering the inflammatory response, involving immune and non-immune cells (myeloid, epithelial, endothelial, etc.) and their products, such as cytokines and chemokines, ROS, neuropeptides, and other mediators [17].

Furthermore, the innate immune system exploits adaptive mechanisms to respond optimally and more vigorously to future repeated infections. Epigenetic and metabolic reprogramming mainly mediates this type of response called trained immunity, which persists for a long period. A recent study in a murine model and in vitro has shown that the western diet (WD) can drive transcriptomic and epigenomic reprogramming of myeloid progenitor cells by the NLRP3 inflammasome pathway, inducing the potentially detrimental and durable effects of myeloid precursor cells’ pro-inflammatory reprogramming in the onset of inflammatory diseases. Moreover, the results of trained immunity on myeloid precursor cells can persist long after switching diet habits toward a healthier diet [17]. Also, other external factors, as stress, can induce training immunity, activating altered and possibly pathological immune responses [18]. Stress can affect intestinal inflammation through the hypothalamic-pituitary-adrenocortical axis and the autonomic nervous system, resulting in pro-inflammatory cytokine production, macrophage activation, intestinal permeability, and microbiota alteration. Observational studies show an association between major stressors, anxiety and depression, and an increased risk of IBD [18].

Cytokines not only regulate intestinal inflammation but are also responsible for extra-intestinal manifestations in IBD. Patients with IBD show an alteration in the balance between pro and anti-inflammatory cytokines. Furthermore, they may have mutations in genes that code for these inflammatory cytokines. For example, the G308A and C511T polymorphisms affect the TNFand IL-1β promoters, respectively, and cause an alteration in the production of these cytokines [19]. Both CD and UC show activation of the humoral immune response. Thus, enhanced humoral IgA and IgG responses to commensal bacteria are relevant characteristics of IBD. Moreover, CD and UC patients show different patterns of Ig-bound bacteria; the first shows an increase in both IgA- and IgG-bound bacteria, which is associated with a severe disease, whereas UC patients show only IgG-bound bacteria [20].

CD is classically associated with an increase in T helper (Th) 1 (Th1) lymphocytes with production of IFN-γ, TNF, and IL-2 in response to IL-12, but also with Th17 cytokines, such as IL-17, IL-21, IL-22, and IL-23. On the other hand, UC patients show an increase in Th2 cytokines, such as TGF-β, IL-10, IL-5, and IL-13 (but not IL-4). Many patients with IBD show an increased expression of IL-17 in the mucosa and sierosa, which is significantly higher in those patients with CD rather than UC. Moreover, Th17-type cytokines (IL-17A, IL-17F, IL-22, IL-26) are over-expressed by T cells in the lamina propria of subjects affected by UC and CD. However, Th-17-related cytokines were correlated with IBD worsening and mucosal deterioration [21]. A study by Gerlach et al. 2014 showed an increase in the expression of IL-9-producing T cells in the mucosa of subjects with UC and several mouse models of oxazolone-induced colitis, suggesting the existence of a subset of Th9 cells involved in the pathogenesis of human ulcerative colitis, as highlighted in the mouse models of experimental colitis. Moreover, the IL-9 receptor is over-expressed in patients with UC [22]. Based on the results from models of oxazolone-induced colitis in which a Th2 profile is expressed, it has been shown that UC has an atypical Th2 response mediated by IL-13 producing NK cells [23,24].

## 2. Immunity and IBD

**Figure 1:**
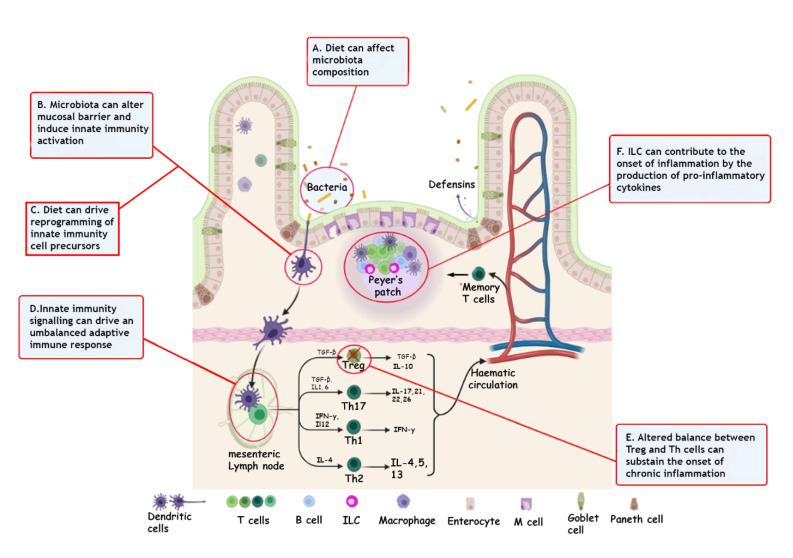
Immune mechanisms involved in the pathogenesis of IBD. (**A**) Diet can affect both microbiota and innate immune cells phenotype toward pro-inflammatory conditions. (**B**) One’s genetic background can make the onset of inflammationmore likely. (**C**) Diet can affect trained immunity of innate cells. (**D**) Gut immune homeostasis depends on the balance between regulatory Tcells and helper T cells. (**E**) Altered signaling between innate and adaptive immunity can shift this balance toward chronic activation of the immune system. (**F**) Cytokines, produced by Th cells, can activate ILC cells, which contribute to sustaining chronic inflammation. Adapted from “Intestinal Immune System”, by BioRender.com (2022). Retrieved from https://app.biorender.com/biorender-templates (accessed on 17 January 2022).

Several studies have made it clear that innate and adaptive immune responses play a crucial role in developing IBD. A malfunction of the intestinal epithelial barrier, one of the main protagonists of innate immunity, largely contributes to intestinal inflammation in patients with UC. Therefore, the immune system must be finely regulated and organized at the intestinal level. It must respond quickly and correctly towards pathogenic bacteria, and, on the other hand, it must maintain tolerance towards commensal bacteria. An imbalance in this close relationship, due to many genetic factors or external stressors, can trigger aberrant inflammatory responses, leading to IBD development (Figure 1). As a further confirmation, several immunological and genome-wide association studies (GWAS) have highlighted the importance of innate immune responses of the mucosa in the pathogenesis of IBD, such as the integrity of the epithelial barrier or the innate microbial detection, the unfolded protein response, and autophagy [25]. The epithelium is mainly formed at the intestinal level by enterocytes and specialized epithelial cells, such as Paneth cells and goblet cells. Tight junctions, adherens junctions, and desmosomes guarantee the functionality of the epithelial barrier [26]. In addition, epithelial cells fully contribute to the defense of the intestine, secreting bactericidal agents, such as defensins, both constitutively and upon recognition of bacterial components by their pattern recognition receptors (PRR). In particular, α-defensins are produced by Paneth cells while β-defensins are produced by epithelial cells [27].

Inflammatory responses against microbial invasion in the intestine occur thanks to the cells of the innate immune system, such dendritic cells (DCsIECs and macrophages that respond to pathogen-associated molecular patterns (PAMPs). In IBD, a high number of DCs at the site of inflammation was observed [28]. These cells activate effector cells, such as CD4^+^ and CD8^+^ T lymphocytes, NK, and NKT cells, and inhibit the activation of regulatory cells. Furthermore, DCs in the intestinal mucosa of patients with CD and UC show high Toll-like type 2 and 4 receptors (TLR2 and TLR4) expression, compared to healthy subjects, and also express high levels of CD40 [28].

The action of DCs leads to the production of high levels of IL-12 and IL-6, altering the mucosa and triggering inflammation. In addition, these cells express the CCR7 receptor, which binds the chemokines CCL19 and CCL21, whichcauses their migration to the T-cell areas of secondary lymphoid organs, further promoting the inflammatory state. This process confirms the close relationship between innate and adaptive immune responses, which, if not finely tuned, contribute to IBD development [29].

Several different proinflammatory cytokines participate inthe progression of IBD. The IL-1 family of cytokines plays a crucial role in UC, where active IL-1β is present at high levels in the colon mucosa and in the CD where IL-18 production is increased [30,31]. This last cytokine seems to be overexpressed in intestinal lesions of patients with CD and represents an essential mediator of Th1 responses, increasing their activity. This does not occur in patients with UC, underlining that the Th2 type immune response plays a predominant role [32,33]. As a matter of fact, in a mouse model of UC, IL-33 (a member of the IL-1 family protects the intestinal epithelium, stimulating mucus secretion) leads to IL-5 and IL-13 overexpression, in line with the Th2 type responses [34].

IL-6 and TNF also play a leading role in the inflammatory response. The former, a pleiotropic cytokine, activates the cytoplasmic transcription factors STAT1 and STAT3 in colon epithelial cells and, together with its soluble receptor, is increased in patients with UC and CD [35]. TNF, on the other hand, plays a fundamental role in the pathogenesis of IBD as it contributes to the increased expression of IL-1β, IL-6, and IL-23, clinically worsening CD and UC. Moreover, serum concentration of soluble receptors for TNF are significantly increased in patients affected by these pathologies. TNF receptor I is overexpressed both in UC and in CD, while TNF receptor II is upregulated in CD patients only. Thus, these two molecules can be exploited in IBD diagnosis [36].

The inflammatory status of the mucosa is attenuated by the immunosuppressive cytokine IL-10, which downregulates the release of proinflammatory cytokines by inhibiting the presentation of the antigen. Unfortunately, there are many inconsistencies in IL-10 expression levels in subjects with IBD. Still, a study in IL-10 gene-targeted mice in chronic ileus colitis has confirmed its role and therapeutic efficacy in several animal models of colitis. Thus, IL-10 may play a therapeutic role in treating IBD [36,37,38].

TGF-β also plays a crucial role in regulating immunological homeostasis and its reduced activity can cause autoimmune disorders in many pathological conditions, including IBD [39]. This cytokine has a double activity in IBD as it stimulates epithelial compensation and fibrosis, and induces tolerance thanks to its immunoregulatory function [40,41].

IL-17, a proinflammatory cytokine present in high concentrations in the inflamed mucosa of IBD patients, also plays an important role. Immunohistochemical studies have shown high levels of IL-17A transcripts in the mucosa of subjects with UC and CD compared to healthy subjects [40,42]. Moreover, high production of IL-17A together with IL-1β, IL-6, IL-21, IL-23, and TGF-β marks mucosal Th17 cells [43]. Subjects with UC and CD overexpress many cytokines related to the Th17 family due to signals from altered microbioma in dysbiosis. Furthermore, it has also been shown that the expression of IL-17 in PBMCs of subjects with UC correlates with the severity of the disease, confirming that this cytokine exerts an inflammatory role mediated by the activation of the STAT3 pathway [40,44]. Th17 lymphocytes need to be finely regulated by regulatory T cells (Treg) to keep the immune responses balanced in the gut. Treg cells express Foxp3 and produce the anti-inflammatory cytokines IL-10 and TGF-β, hence playing anti-inflammatory activities. Furthermore, studies in mouse models have shown that Treg cells can extinguish inflammation in the intestine, thanks to the release of IL-10 [45,46]. This cell type is also almost totally decreased in the peripheral blood of patients with active IBD, unlike subjects with quiescent IBD, underlining its importance in avoiding the progression of the disease. In physiological conditions, both Th17 and Treg cells are represented in the intestinal mucosa, where they perform protective functions against pathogenic microorganisms, thus limiting the responses of effector T cells. The alteration of Th17 *versus* Treg balance is influenced by the intestinal microbiome, which plays a crucial role in the onset of IBD. In fact, in cases of intestinal dysbiosis, susceptibility to IBD increases [47,48,49]. This finding correlates with the potential therapeutic effects in chronic intestinal inflammation mediated by Treg cells [50,51]. Similarly, possible new therapies for IBD include the neutralization of proinflammatory cytokines or their receptors by monoclonal antibodies (mAbs). For example, studies on mouse models have shown that mAbs directed against IL-12 and IL-23 p40 can improve the severity of colitis; similarly, mAb against IL-21 leads to a downregulation of the infiltration of T cells in the colon and of some proinflammatory cytokines, such as IL-6 and IL-17A in the inflamed intestine in mice with colitis induced by dextran sulfate sodium (DSS) [52,53].

Other effectors of innate immunity that significantly contributes to maintaining the integrity of the intestine are innate lymphoid cells (ILC). ILCs participate in thedefense against intestinal infections, regulate adaptive immunity, play an essential role both in the formation of lymphoid tissues and in homeostasis, and tissue regeneration [54,55]. They have been identified in different parts of the body: blood, tonsils, thymus, liver, intestine, lung, skin, uterus, and bone marrow. They significantly contribute to the state of health or disease of the gastrointestinal tract of mammals, mainly located in the intestinal barrier and the lamina propria of the small intestine and colon. Here they perform effector functions mediated by the production of cytokines, by cytolytic activity typical of T lymphocytes, but different from those of CD3^+^T lymphocytes, and direct cell–cell interactions with stromal cells and other immune cells. Three main characteristics define them: the absence of clonally distributed antigen recognition receptors generated by gene recombination, the lack of phenotypic markers typical of myeloid and dendritic cells, which is why they are defined as negative lineage cells (Lin-), and lymphoid cell morphology [56,57]. Based on the expression of specific transcription factors and the different cytokine profiles, it is possible to distinguish three different subtypes of ILC. The ILCs of group 1 (ILC1) express the transcription factor T-bet, respond to IL-12, IL-18, and IL-15, and produce IFN-γ and TNF. This group also includes T-bet^+^Eomes^+^ Natural Killer (NK) cells and the T-bet^+^Eomes^−^ILC1. Group 2 ILCs (ILC2) express the transcription factor GATA-3, respond to IL-25, IL-33 and TSLP, and produce IL-5, IL-13, IL-9, and amphiregulin. They play an important role against helminthic infections and in the pathogenesis of asthma and allergies. Group 3 ILCs (ILC3), which express the transcription factor RORγ-t, respond to IL-1β and IL-23, and produce IL-22 and IL-17A, cytokines that play a crucial role in the development of lymphoid tissues and the balance between the host and microbes at the level of the mucosal surfaces [58]. This group also includes Lymphoid Tissue Inducer (LTI) cells, which are involved in forming secondary lymphoid tissues, such as lymph nodes and Peyer’s patches in the intestine [57,59]. The main immunopathogenic processes are described in Figure 1.

Diet is a crucial factor that plays a pivotal role in regulating immune responses, influencing the various cell types mentioned above, and consequently the expression of cytokines that negatively affect the prognosis of IBD.

## 3. Nutrition and IBD

**Figure 2:**
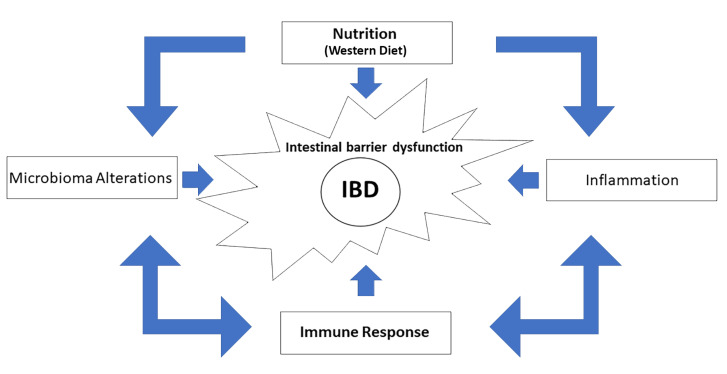
Nutrition is critical to the development and progression of IBD. Eating habits influence the risk of IBD through numerous and complex mechanisms affecting the intestinal microbiota and leading to the inflammation of the intestine. Thus, causing deregulated immune responses that worsen the prognosis of IBD.

Extensive epidemiological evidence proves the importance of nutrition on the development, symptoms, and prognosis of IBD. A particular dietary regimen can strongly influence IBD risk through multiple mechanisms. Among these, diet habits modify the intestinal microbiota and, consequently, affect the immune response leading to changes in the activity of the immune system [14,15,17] (Figure 2). Industrialization, together with the improvement of health conditions, antibiotics, and the increasing consumption of fats and sugars deriving from typical WD, all change the composition and metabolic activities of the human intestinal microbiome. Little is known on how intestinal bacteria responds to dietary changes. Two studies have shown that diet significantly changes the gut microbiome in inbred mice in just 24 h [60,61]. On the other hand, it takes humans much longer to experiencesignificant and durable changes [62].

Various studies have shown that the variations in the composition of micro- and macronutrients from the first years of life and afterward can favor an anti-inflammatory or pro-inflammatory status [63,64]. 

The inflammatory status, also related to diet, appears to be the tip of the balance that plays a crucial role in determining the evolution of IBD in affected patients or those genetically predisposed. For example, the Western lifestyle, characterized by high-caloric diets with excessive consumption of fats and carbohydrates, shifts the balance towards an inflammatory state. Eating habits in industrialized countries are characterized by high concentrations of pro-inflammatory omega-6 fatty acids, at the expenses of purely anti-inflammatory omega-3 fatty acids [65]. These findings explain why diet is becoming increasingly crucial in developing or improving IBD (Table 1).

The WD, characterized by a high consumption of fats and carbohydrates, shifts the inflammatory balance towards a purely pro-inflammatory status. Meanwhile the habitual consumption of omega-3 fatty acids, fibers, and vegetables typical of a Mediterranean lifestyle represent a substrate for the (SCFA) by intestinal microbes that extinguish inflammation by shifting the balance towards an anti-inflammatory status.

The first items of evidence supporting the influence of a particular dietary regimen on human health concerns the microbiome’s composition. Intestinal dysbiosis, which involves an imbalance in the composition of the families of microbes in the intestine, is a hallmark of IBD. Lewis et al. showed that typical WDs are closely associated with poor microbiome diversity [66]. Dysbiosis is also related to increased invasive pathogenic microbial species as adherent-invasive *Escherichia coli* [67]. A recent study also showed that in IBD, there are differences in the function of intestinal microbes themselves, which may be more marked than the differences between species [71]. These changes can cause inflammation of the mucosal barrier, another hallmark of IBD [66]. However, it is the composition of the nutrients that interacts with the immune defenses of the intestinal mucosa and inflammatory and non-inflammatory cells, that influences their responses. For example, the fibers and starches found in vegetables and fruit, abundant in the Mediterranean Diet, are a substrate to produce Short-Chain Fatty Acids (SCFA) by intestinal microbes. These include butyrate, a waste element in the digestion of dietary fibers by the intestinal microbiome, which plays a crucial role in the immune system, stimulating the production of Tregs in the lamina propria and inhibiting the transcription of inflammatory cytokines. It also represents a critical factor in epithelial homeostasis: low levels of butyrate and dietary fiber, in general, accelerate the catabolism at the level of the mucosa, leading to an increased intestinal permeability which is more susceptible to potential luminal pathogenic bacteria [72,73,74].

Other studies have shown how much diet affects immune responses. For example, the classic WD rich in sugars and fats causes the upregulation of TNF and IFN-γ expression in mice with a consequent decrease in the Tregs of the colon. This scenario generates a proinflammatory intestinal environment that aggravates dysbiosis [75,76]. Furthermore, in a mouse model, excessive fat consumption may favor gram-negative bacteria (e.g., Enterobacteriacee) proliferation, leading to an increase inendotoxin production, such as bacterial lipopolysaccharide (LPS). The increased amounts of LPS causes an increase in IL-1β, TNF, IL-6 production, and the activation of the NF-κB pathway via TLR4 in the colon, causing inflammasome-dependent acute intestinal inflammation [77]. In another mouse model, high-fat-diet induces an increase of TNF in the colon and in turn activates Wnt signaling, suggesting a molecular mechanism for obesity-associated colorectal carcinogenesis [78]. Also in humans, a fatty rich diet can induce a continuous alteration of the microbiome, thus leading to lower production of anti-inflammatory and immunoregulatory molecules, such as butyrate, whose reduced concentration contributes to the increased inflammation of the intestine [79]. The microbiome alteration was evidenced in patients with CD who showed an increase in enterobacteria and a decrease in Clostridiales [80,81]. Reduced microbial diversity is also evident in subjects with UC but less marked than in patients with CD [82]. A cohort study highlighted the differences in the microbiome between IBD patients and healthy people in four distinct European countries by analyzing their fecal samples [83]. Some microbes, such as Listeria monocytogenes or Toxoplasma gondii, have been shown to activate ILC1 and Th1 cells, modulating cytokine secretion, such as IFN-γ and TNF, which are essential against some intestinal pathogens [84]. On the contrary, Clostridia strains promote the accumulation of Foxp3^+^Treg cells in the intestine, thus regulating inflammatory responses, including those induced as in experimental colitis in mice [85,86]. Desai et al. pointed out that a low-fiber, high-fat diet in mice, typical of WD in humans, causes alterations in the intestinal microbes that exploit the glycoproteins of the mucosal layer as the primary source of nutrients, triggering erosion of the mucus barrier of the colon, the mainline of defense against enteric pathogens [74]. Furthermore, some microbes, such as bifidobacterial and segmented filamentous bacteria, in the intestine directly activate immune cells that promote inflammation, such as Th17 cells. Their decrease causes a reduction in the severity of colitis in mice [87,88].

Macronutrients most often studied to better understand the association between diet and IBD are polyunsaturated fatty acids (PUFA), including linoleic acid (LA) and α-linolenic acid (ALA), which belong respectively to omega-6 and omega-3, and are called essential fatty acids as the human body does not synthesize them. LA and arachidonic acid are precursors of purely pro-inflammatory eicosanoids, while ALA and docosahexaenoic acid (DHA) are precursors of eicosanoids with anti-inflammatory properties [68,89]. High quantities of omega-6 fatty acids are found in vegetable oils, such as sunflower oil and in margarine, while omega-3 fatty acids are found in fatty fish and liver oil cod, while ALA is found in linseed oil and green leafy vegetables [14]. IBD is characterized by an increase in the omega-6/omega-3 ratio which may be related to the increased incidence of CD as indicated by a Japanese study [69,90]. Furthermore, another study showed a doubled risk of developing UC in subjects with high intakes of omega-6 PUFA LA and a reduced risk of 77% in patients with higher dietary intake of DHA acid [91]. It further confirms a study on nurses’ health where the long-term, high intake of omega-3 PUFA was associated with a lower risk of UC than the increased consumption of trans-unsaturated fatty acids related to increased risk [92].

Fatty acids affect the immune system in several ways, such as the production of anti- and proinflammatory mediators, the modification of intracellular lipids, and the activation of nuclear receptors. The immunomodulatory properties of omega-3 PUFA, for example, concern the production of bioactive fat derivatives. Omega-3s regulate the production of pro-inflammatory molecules, such as prostaglandins, leukotrienes, and thromboxane, and control the inflammatory response. For this reason, they are defined as Specialized pro-resolving Mediators (SPMs), equipped with anti-inflammatoryand restoring homeostasis properties, downregulating proinflammatory cytokines, and upregulating anti-inflammatory ones. They also activate phagocytes towards debris and lifeless cellular components, decreasing oxylipins’ levels, which increase inflammation [91,92,93,94].

In mice, the Omega-3 PUFA also inhibit M1 polarization of activated macrophages that release TNF and IL-1β, and increase the phagocytic capacity of neutrophils. In in vitro tests, adding DHA to separated peritoneal neutrophils improved their phagocytic and fungicidal ability by 35%. Moreover, adding eicosapentaenoic acid (EPA) or DHA to polymorphonuclear leukocytes from goats in culture, enhanced their phagocytic capacity against *Escherichia coli*. Along the same lines, supplementing 10 people with fish oil comprising 26% EPA and 54% DHA every day for a total of 60 days led to a 62% increase in neutrophilic phagocytosis [70,95]. Fatty acids also interact with TLRs, particularly with TLR2 and TLR4, on leukocytes. For example, saturated fatty acids interact with these TLRs by increasing COX-2 expression and ERK phosphorylation. They also activate other proinflammatory pathways concerning the NLRP3 inflammasome with cytokine production, such as IL-1β and IL-18. On the other hand, Omega-3 and DHA suppress COX-2 and ERK phosphorylation and the production of IL-17 from Th17 cells with a consequent reduction of STAT-3 phosphorylation. STAT-3 dephosphorylation leads to the restoration of homeostasis and reduces intestinal inflammation [96]. Moreover, a study evaluated the influence of an increased dietary intake of omega-3 PUFA and a reduced intake of omega-6 PUFA in a cohort of subjects with IBD. Remission at a 12-month followup was associated with a higher ratio of omega-3/omega-6 PUFA in the red blood cell membrane compared to the ratio observed in patients who had relapsed [97]. Therefore, the activity of the disease can be modulated by diet; high consumption of fibers and an increased ratio of omega-3/omega-6 PUFA reduce the risk of exacerbation in both forms of IBD.

However, other studies indicate no association between fat intake and the increased risk of IBD, such as UC. For example, a Japanese case-control study showed a positive association between excessive consumption of omega-3 PUFA and the risk of CD. According to Sakamoto et al., this could be explained on the ground that the increased consumption of oily fish increases the overall fat intake, and this could increase the risk of developing IBD [98,99]. These conflicting results could be due to the different metabolism of PUFA in each patient, suggesting that specific enzymes metabolize all dietary fatty acids. These enzymes are genetically regulated; in particular, a polymorphism in the CYP4F3 locus positively modifies the association between omega-3 and omega-6 PUFA in the diet of UC patients [100]. Therefore, it seems evident that in addition to genetic factors, nutrition affects intestinal homeostasis based on multiple mechanisms and relationships between IECs, microbiome, and immune cells. Related to this, SCFA are ligands of G protein-coupled receptors, such as GPR43, a receptor involved in the activation and recruitment of neutrophils. Based on the composition of the intestinal microbiome and the integrity of the mucosal barrier, these cells can infiltrate the wall, contributing to the development of inflammatory processes in the gastrointestinal tract and subsequently lead to the production of reactive oxygen species and chemokines that amplify inflammation and affect the mucous membrane [101].

For these reasons, over the years, there have been numerous dietary approaches analyzed in the clinic to improve IBD symptoms. However, these eating plans have significant restrictions and are therefore used in more severe patients or, for example, in children where steroid therapy could have significant repercussions on their growth [102]. In addition, steroids do not guarantee the healing of the intestinal mucosa [103].

Without a shadow of a doubt, among the most used dietary interventions, exclusive enteral nutrition (EEN) stands out in which the nutritional needs are fully guaranteed through liquid formula orally or through the use of the nasogastric tube for about seven weeks, guaranteeing the same remission equivalent to steroids in the pediatric population [15,104,105]. EEN helped induce and maintain remission in adults and children with CD and was more effective in patients with CD than with UC [106,107]. The EEN includes elementary and polymeric feeds, all lactose and gluten-free with low fat and therefore easily digestible and water-soluble, guaranteeing anti-inflammatory effects and mucosal healing [108,109,110].

A food-based diet was also evaluated along the same lines as the EEN to improve the compliance of CD patients, excluding some nutrients, such as gluten, lactose, and alcohol, called the Ordinary Food Diet (CD-TREAT), which showed a reduction of inflammatory markers in 12 weeks [15,111].

The specific carbohydrate diet (SCD), based on eliminating certain complex carbohydrates, sugar, many dairy products, and all processed foods, also showed reduced inflammation in 12 weeks despite needing more studies to investigate. Although these foods are poorly absorbed in the intestine, they can have a pro-inflammatory role [14,15,112].

The most recently proposed and promising diet for patients with CD is the Crohn’s Disease Exclusion Diet. It provides a complete food diet with fruits, vegetables, meat, and complex carbohydrates, minimizing all those nutrients that could affect the mucosa, increasing intestinal permeability [113,114]. In addition, this nutritional plan excludes the consumption of animal fats, limiting specific cuts and types of meat, gluten, emulsifiers, sulphites, and even some monosaccharides on the same line as the EEN. This new approach has shown promise, especially in patients who fail classical biological anti-TNF therapy [114].

Therefore, it is increasingly evident that the use of a targeted diet to prevent and treat IBD without side effects can be a winning therapeutic strategy. However, to achieve this goal, it is necessary to involve doctors, nutritional biologists, and even psychologists who, working as a team, can help and support patients throughout the nutritional process to obtain a partial or total remission of the symptoms.

## 4. Conclusions

The results of several studies indicate that nutrition plays a crucial role in the pathogenesis of IBD. However, nowadays it is still difficult to understand the specific mechanisms that the various nutrients bring to improve or worsen the disease state and/or the symptoms. This is because too many factors are involved in the development of IBD: microbioma, IECs, immune cells and their products, macro- and micronutrients, and metabolites deriving from them. All these factors also interact and influence each other according to a series of complex mechanisms that require other, more in-depth studies. Nevertheless, particular dietary behaviors far from those typical of WD may be useful not only in preventing IBD in genetically predisposed subjects but, most importantly, in delaying its possible onset.

## Figures and Tables

**Table 1:** Effect of macronutrients in modulating immune responses.

	Cytokines, Immune Cells and Microbioma						
	TNF	IL-1β	IL-6	M1 Macrophages	Treg	GRAM Negative Bacteria	References
High consumption of fats and sugars	+	+	+	+	−	+	[14,15,17,25,29,30,36,63,66,67]
Regular consumption of omega-3 PUFA, fibers and vegetables	−	−	−	−	+	−	[14,15,50,51,65,66,68,69,70]

## Data Availability

Not applicable.
